# Complete Genome Sequence of an Avian H1N1 Influenza Virus Strain Isolated from Migratory Birds in the Republic of Korea

**DOI:** 10.1128/genomeA.00356-17

**Published:** 2017-05-25

**Authors:** Bon-Sang Koo, Hye Kwon Kim, Woonsung Na, Daesub Song, Doo-Jin Kim, Sun-Woo Yoon, Dae Gwin Jeong

**Affiliations:** a Infectious Disease Research Center, Korea Research Institute of Bioscience & Biotechnology, Daejeon, Republic of Korea; b Department of Pharmacy, College of Pharmacy, Korea University, Sejong, South Korea; c University of Science and Technology (UST), Daejeon, Republic of Korea

## Abstract

Here, we report the complete genome sequence of an H1N1 avian influenza virus (AIV), which was isolated from the feces of migratory birds in the Republic of Korea during the winters of 2014 and 2015. Full-genome sequencing and phylogenetic analysis revealed that all genome segments belonged to the Eurasian lineage.

## GENOME ANNOUNCEMENT

An avian H1N1 influenza virus strain, A/wild bird/Korea/SK14/2014, was isolated from feces samples of migratory birds. For genetic analysis, total viral RNA was extracted using a QIAamp viral RNA minikit (Qiagen, Germany), and cDNA was synthesized using SuperScript III reverse transcriptase (Invitrogen, CA) with the Uni-12 primer (5′-AGCRAAAGCAGG-3′), as per the manufacturer’s protocol. PCR products were amplified with universal primer sets targeting the full genomes of influenza virus using the Phusion master mix kit (Thermo Fisher Scientific, MA) (1). Nucleotide sequences were obtained by direct sequencing using the ABI3730XL DNA analyzer (Cosmo Genetech, South Korea). The sequences were manipulated in the BioEdit program (http://www.mbio.ncsu.edu/bioedit/bioedit.html) and assembled in CLC Sequence Viewer 6.7. The multiple alignments of full coding nucleotide sequences were performed by MUSCLE algorithm. Phylogenetic analysis based on nucleotide sequences was constructed with the neighbor-joining method with 1,000 replicates in Molecular Evolutionary Genetics Analysis (MEGA), version 6.06 (2).

The complete genomic lengths of 8 segments containing the polymerase basic 2 (PB2), PB1, polymerase acidic (PA), hemagglutinin (HA), nucleoprotein (NP), neuraminidase (NA), nonstructural (NS), and matrix (M) genes represented 2,280, 2,274, 2,151, 1,701, 1,497, 1,410, 982, and 838 nucleotides, respectively. In HA protein, the cleavage site possesses only a single basic amino acid (PSIQSR↓GLF), which is characteristic of low-pathogenicity avian influenza virus (AIV) (3). Interestingly, the 2 amino acid residues within the receptor binding site of the HA were 133K and 226Q (H3 numbering) mutated, which was related to enhanced binding preference to the α2-6 receptor and its favoring of human-like receptors (4, 5). The substitution of amino acid motifs in the PB2 gene (positions E627 and D701), which is associated with increased transmissibility and/or pathogenicity in mammalian hosts, was not found. In addition, no mutations of motifs were found to be associated with oseltamivir resistance on the NA position at H275 (6) and amantadine resistance on the M gene (7). In other segments, including the PA, NP, and NS proteins, there were no specific mutations of motifs which were related to increased infectivity and pathogenicity against mammalian species.

Our study is meaningful for furthering the understanding of molecular evolution in the Eurasian lineage of H1N1 avian influenza virus and will facilitate future investigations of the epidemiology of this virus.

### Accession number(s).

The genome sequence of the avian H1N1 influenza virus, A/wild bird/Korea/SK14/2014, was deposited in GenBank under the accession numbers KX066868 to KX066875.
